# Recent Progress in (Photo-)-Electrochemical Conversion of CO_2_ With Metal Porphyrinoid-Systems

**DOI:** 10.3389/fchem.2021.685619

**Published:** 2021-07-16

**Authors:** Dženeta Dedić, Adrian Dorniak, Uwe Rinner, Wolfgang Schöfberger

**Affiliations:** ^1^ Institute of Organic Chemistry, Johannes Kepler University Linz, Linz, Austria; ^2^ IMC Fachhochschule Krems, Krems an der Donau, Austria

**Keywords:** catalysis, carbon dioxide reduction, metal complexes, corroles, porphyrins, phthalocyanines, electrocatalysis

## Abstract

Since decades, the global community has been facing an environmental crisis, resulting in the need to switch from outdated to new, more efficient energy sources and a more effective way of tackling the rising carbon dioxide emissions. The activation of small molecules such as O_2_, H^+^, and CO_2_ in a cost—and energy-efficient way has become one of the key topics of catalysis research. The main issue concerning the activation of these molecules is the kinetic barrier that has to be overcome in order for the catalyzed reaction to take place. Nature has already provided many pathways in which small molecules are being activated and changed into compounds with higher energy levels. One of the most famous examples would be photosynthesis in which CO_2_ is transformed into glucose and O_2_ through sunlight, thus turning solar energy into chemical energy. For these transformations nature mostly uses enzymes that function as catalysts among which porphyrin and porphyrin-like structures can be found. Therefore, the research focus lies on the design of novel porphyrinoid systems (e.g. corroles, porphyrins and phthalocyanines) whose metal complexes can be used for the direct electrocatalytic reduction of CO_2_ to valuable chemicals like carbon monoxide, formate, methanol, ethanol, methane, ethylene, or acetate. For example the cobalt(III)triphenylphosphine corrole complex has been used as a catalyst for the electroreduction of CO_2_ to ethanol and methanol. The overall goal and emphasis of this research area is to develop a method for industrial use, raising the question of whether and how to incorporate the catalyst onto supportive materials. Graphene oxide, multi-walled carbon nanotubes, carbon black, and activated carbon, to name a few examples, have become researched options. These materials also have a beneficial effect on the catalysis through for instance preventing rival reactions such as the Hydrogen Evolution Reaction (HER) during CO_2_ reduction. It is very apparent that the topic of small molecule activation offers many solutions for our current energy as well as environmental crises and is becoming a thoroughly investigated research objective. This review article aims to give an overview over recently gained knowledge and should provide a glimpse into upcoming challenges relating to this subject matter.

## Introduction

The aim of this review is to give an overview of different metal porphyrinoid electrocatalysts and to outline their ability to activate and reduce CO_2_. In order to explain the difficulties of this electrocatalytic process, we discuss the mechanisms of CO_2_ reduction and show current of such catalysts. The most recent findings in catalyst development, in respect to different metal ions as central atoms and ligand systems used are discussed in detail.

### Porphyrins, Corroles, and Phthalocyanines

**FIGURE 1:**
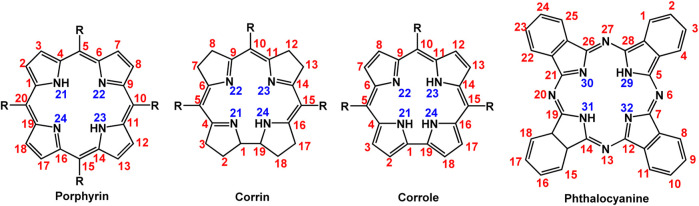
General structures of tetrapyrrole molecules.

Porphyrins are naturally occurring cyclic macromolecules, which consist of four pyrrole rings connected *via* methine groups. Corroles differ from porphyrins as one of the methine groups is missing. In phthalocyanine macrocycles, four isoindole groups are linked together *via* nitrogen bridges. Figure 1 presents the general structures of porphyrin, corrole, and phthalocyanine with IUPAC numeration. The positions 5, 10, and 15 (or 5, 10, 15, and 20 in case of porphyrin) are called meso-positions (Moss, 1988; Sorokin, 2013; Zhang et al., 2017a; Barata et al., 2017).

Porphyrin and corrin derivatives are responsible for many important biochemical processes and often play an important role in living organisms, mainly serving as biocatalysts. These macrocycles serve as ligands as they tend to form coordinative bonds between the nitrogen atoms of the pyrrole units and transition metal ions such as Fe, Ni or even alkaline earth metals such as Mg.

A well-known example of a naturally occurring tetrapyrrole system is hemoglobin. The central atom of the compound (iron) forms coordinative bonds with oxygen and enables the oxygen transport in blood. Another example is chlorophyll, the green pigment present in plants, algae, and some bacteria. This complex of chlorin (reduced structure of porphyrin) with magnesium ion is responsible for the conversion of solar energy into chemical energy during photosynthesis. Cofactor F_430_ and cobalamin (vitamin B_12_) are other examples of naturally occurring tetrapyrroles. Despite similar properties, corroles, and phthalocyanines have not been isolated from natural sources so far (Shooling-Jordan and Cheung, 1999; Layer et al., 2010; Sorokin, 2013; Sawicki et al., 2015).

Coordinatively bonded tetrapyrrole systems strongly absorb electromagnetic radiation in the in the visible range. These compounds typically are highly stable in the solid and solution states, which simplifies characterization and structure elucidation and furthermore provides advantages for mechanistic investigations. Tetrapyrrol systems are easily modified and/or functionalized which paves the way for sophisticated applications in various ways. Changing substituents at meso- and β-positions as well as introducing additional functional groups at the second coordination sphere enables precise tuning of chemical and physical properties. Such modifications also include metalation, synthesis of macrocycles from previously functionalized educts or postfunctionalization of the inner core on peripheral positions. Examples of such modifications are halogenation, formylation, carboxylation, nitration, amination, sulfonation, oligomerization, chlorosulfonation, nucleophilic aromatic substitution or metal catalyzed cross-coupling reactions (Paolesse et al., 2001; Vicente and Smith, 2014; Barata et al., 2017; Hiroto et al., 2017; Orłowski et al., 2017; Nardis et al., 2019).

During the metalation process, tetrapyrrol systems are deprotonated at the inner core, leading to a four-coordinated square-planar configuration of the complex. Corroles differ from porphyrins or phthalocyanines by the number of hydrogen atoms in the inner core, which leads to different behavior during the metalation process. Deprotonated corrole binds metal ions with three bonds, while porphyrin or phthalocyanine chelates to metal ions with only two bonds. The trianionic ligand derived from corrole stabilizes metal ions more effectively in high oxidation states than their analogs with increased ring size. This has a positive effect on the formation and also the cleavage of coordinative bonds which will be discussed in subsequent sections of this chapter (Hiroto et al., 2017).

Porphyrins and corroles substituted at meso and β-positions are interesting for a variety of different applications. Fine-tuning of optical, electronic, and biochemical properties of these coordination compounds is generally achieved *via* the installation of different functional groups whereas the nature of the functionality and the position of the modifications defines the properties of the final product. (Pinto et al., 2016; Barata et al., 2017).

Different routes towards the preparation of corroles and porphyrines have been reported. Generally, the condensation of pyrroles with the requisite aldehydes are used to establish the macrocyle. This reaction, typically catalyzed by acids, is then followed by oxidation with *p*-chloranil or DDQ to complete the process (Lindsey et al., 1987; Vicente and Smith, 2014; König et al., 2016; Pinto et al., 2016). Quite often, yields are low as the reactivity of intermediates increases with growing chain length, resulting in the formation of undesired side products (Blumenfeld et al., 2015). Also, the purification is often tricky and difficult to perform on larger scale. Summarizing, very often, the complicated preparation of these compounds strongly limits their potential use in the industry.

A general approach based on the strategy outlined above was reported by Lindsey’s group in 1986. Various meso-substituted porphyrins were obtained in high-overall yield when the condensation reaction of pyrrole and an aldehyde was carried out in high dilution (Lindsey et al., 1987). Initial attempts towards the preparation of corroles were quite limited in respect to the complexity of the desired final products (Paolesse et al., 1994).

A great improvement in the synthesis of meso-substituted corroles was reported by Koszarna and Gryko in 2006. By employing biphasic reaction mixtures, the authors were able to drastically improve on previously reported yields. Simultaneously bilanes, which exhibit lower solubility, precipitated from the reaction mixture, preventing the product from undergoing unwanted side reactions and oligomerization. The protocol furthermore enabled the synthesis of corroles, substituted with strongly electron-donating groups, in good overall yield. Previously reported methods did not allow the synthesis of this important class of modified corroles (Koszarna and Gryko, 2006; König et al., 2016).

Despite the progress in the preparation of tetrapyrrole systems, scale up is still economically challenging, as all protocols rely on the use of large amounts of solvents. The solution to this problem may be the use of mechanochemistry. It has been demonstrated, that solvent-free synthesis of porphyrine in ball mills is possible, giving hope for the potential industrial-scale production of this class of compounds (Shy et al., 2014).

Among other tetrapyrrolic systems, phthalocyanines seem to be the best-suited catalysts for small molecule activation (Boutin et al., 2020). These compounds provide several advantages over corroles and porphyrins. Precursors and starting materials required for the syntheses are often inexpensive, which allows for the large-scale preparation of these compounds and the preparation and the purification of the products is well-established. Similar to corroles and porphyrins, the synthesis of both symmetric as well as asymmetric phthalocyanines has been described. Fine-tuning of solubility properties in a variety of common organic solvents as well as water is possible *via* the addition and incorporation of appropriate substituents (Sakamoto and Ohno-Okumura, 2009; Sorokin, 2013; Nemykin et al., 2014; Denekamp et al., 2019; Araújo et al., 2020).

### Electrocatalytic Reduction of CO_2_

**FIGURE 2:**
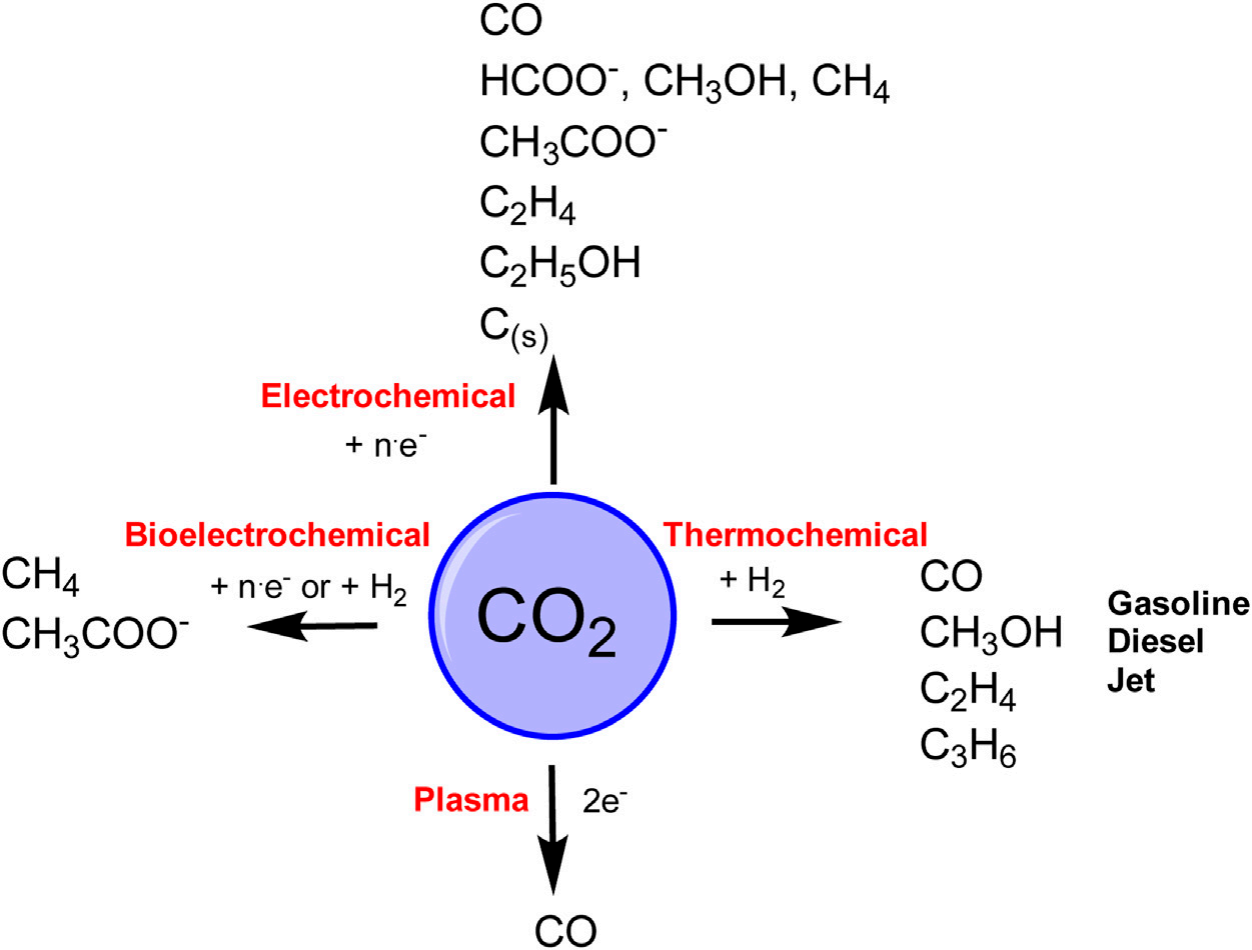
Electrochemical-, bioelectrochemical-, thermochemical- and Plasma reduction of CO_2_ (e^−^—direct pathway, H_2_—indirect pathway).

**FIGURE 3:**
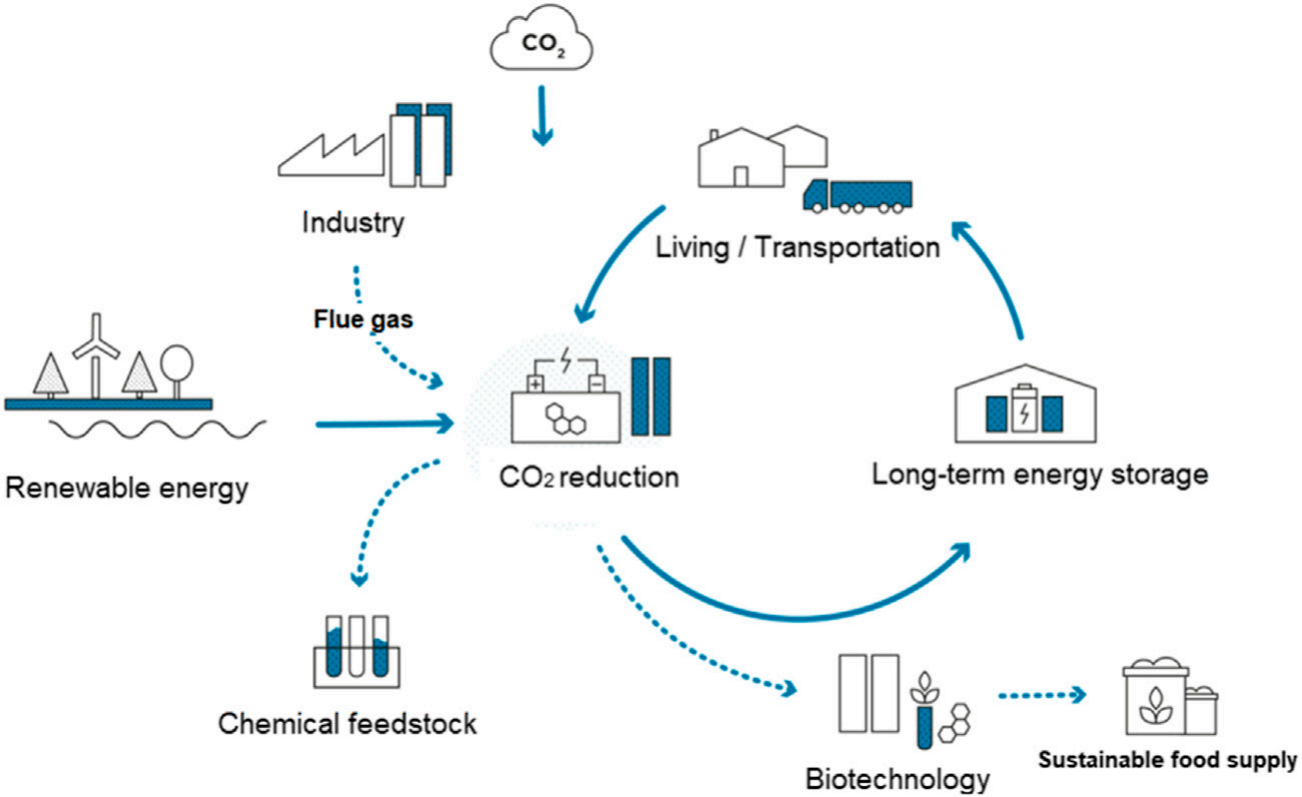
CO_2_ Utilization—a sustainable and climate-friendly cycle.

Global warming and the emissions of climate-relevant gases such as carbon dioxide have been a major area of discussion in recent years and decades. Comprehensive solutions are required with respect to CO_2_ management in order to maintain the maximum level of global warming below a threshold of +1.75°C. Since the reduction of CO_2_ emissions alone will probably not be sufficient, other methods such as the storage and recycling of CO_2_ must also be increasingly addressed. Such concepts of CO_2_-recycling include thermochemical-, (bio)-electrochemical reduction or by plasma-driven catalytic reduction methods (Villano et al., 2010; Bajracharya et al., 2017; Yuan et al., 2019; Bogaerts and Centi, 2020; Liu et al., 2020) of carbon dioxide to fuels such as methane, methanol, and ethanol (Figure 2). If the electrical energy required for this conversion was solely generated from renewable sources, a completely sustainable and climate-friendly cycle would become possible (Figure 3). The electrical energy required for the reduction of CO_2_ could be directly obtained *via* light harvesting using semiconductors. As the storage of electrical energy remains an unsolved problem to date, the direct conversion of CO_2_ to value-added products also could serve as solution to this tricky challenge. In this respect, wind or solar energy surpassing the capacity of the power grid could be used for the production of methanol or ethanol. With the conversion of one ton of CO_2_ equivalents, almost 1,400 kWh of energy could be stored under ideal conditions. If necessary, the methanol can then be used again, for example, as fuel in a direct methanol/ethanol fuel cell (DMFC).

To date, efforts to selectively convert CO_2_ into methanol, ethanol or long-chain hydrocarbons continuously and at high rates have so far failed because the electrochemical reduction of CO_2_ is associated with several problems: 1) The solubility of CO_2_ in water is low and thus leads to low reaction rates. 2) The reduction of CO_2_ is a reaction that takes place in several steps and leads to a number of possible organic compounds. The reaction mechanism is highly dependent on the experimental conditions and it is difficult to control the selectivity of the process (Zhang et al., 2017a). The reduction of CO_2_ usually takes place at potentials at which the hydrogen evolution occurs. This side reaction greatly reduces the efficiency of the process (Sorokin, 2013). Additionally, the electrocatalytic activity of the respective metal catalysts might decrease significantly with time. Therefore, a thorough determination of the stability of the respective catalyst system is a necessity.

Despite the importance of the overall process, and the effort devoted to this field, answers to the questions outlined above have not yet found.

Although the reduction of CO_2_ and light-induced water-splitting is possible, these processes require the application of novel catalysts. The reduction of CO_2_ typically affords complex product mixtures. As nature has clearly demonstrated the feasibility of this approach, porphyrinoid-based catalysts seem highly suitable as catalysts as such protocols mimic photosynthesis (Mele et al., 2014; Zhang et al., 2017a; Nardis et al., 2019; Boutin et al., 2020; Min Park et al., 2020).

## Reduction of Carbon Dioxide—Problematic Issue

The modern lifestyle of mankind with its continued combustion of fossil fuels has resulted in a dramatic increase of green-house gases such as CO_2_. The increase of CO_2_ in our atmosphere results in global warming and climate change (Aresta et al., 2013; Qiao et al., 2014; Álvarez et al., 2017). In 2018, the combustion of fossil fules led to an increase of the CO_2_-level in the atmosphere of 33.1 GT. In the same year, the emission was further increased by 1.7% (IEA, 2019). Ideally, the excess of CO_2_ produced by mankind should be converted in re-usable chemicals to close the circle and provide environmental stability (Qiao et al., 2014).

With the growing importance of capturing and managing man-made CO_2_, different technologies have been evaluated. While one approach envisages the capture of CO_2_ with subsequently sequester it geologically, another approach explores the conversion of CO_2_ *via* chemical methods, such as photo—and electrocatalytical protocols, using homogeneous or heterogeneous catalysts (Aresta et al., 2013; Khezri et al., 2017). The latter approach would allow the production of low-carbon fuels and other important synthons for industrial processes, such as carbon monoxide (CO), formic acid (HCOOH), acetic acid (CH_3_COOH), methane (CH_4_), ethylene (C_2_H_2_), oxalate (C_2_O_4_^2−^), formaldehyde (HCOH), and methanol (CH_3_OH) or ethanol (CH_3_CH_2_OH).

In the last years, the electrochemical CO_2_ reduction has attracted great attention and many research groups entered the quest for a sustainable economic and ecological solution of this ever-growing problem. The process of CO_2_ reduction is interesting as the process is easily controllable *via* temperature and electrode potentials. Supporting electrolytes can be fully recycled, electricity for the processes can be obtained from renewable sources, such as geothermal, solar or wind. Furthermore, the electrochemical units required for the conversion of CO_2_ to value-added products are compact and do not require much space (Qiao et al., 2014; Khezri et al., 2017). Although, these points sound very positive, some challenges remain. Major points are high-overpotentials, due to slow reaction kinetics, stability of catalysts and low product selectivity. As a result, one of the biggest challenges is consequently the development of new stable and selective electrocatalysts (Enthaler et al., 2010; Kondratenko et al., 2013; Qiao et al., 2014; Khezri et al., 2017).

### Reactivity and Activation of Carbon Dioxide

**SCHEME 1:**
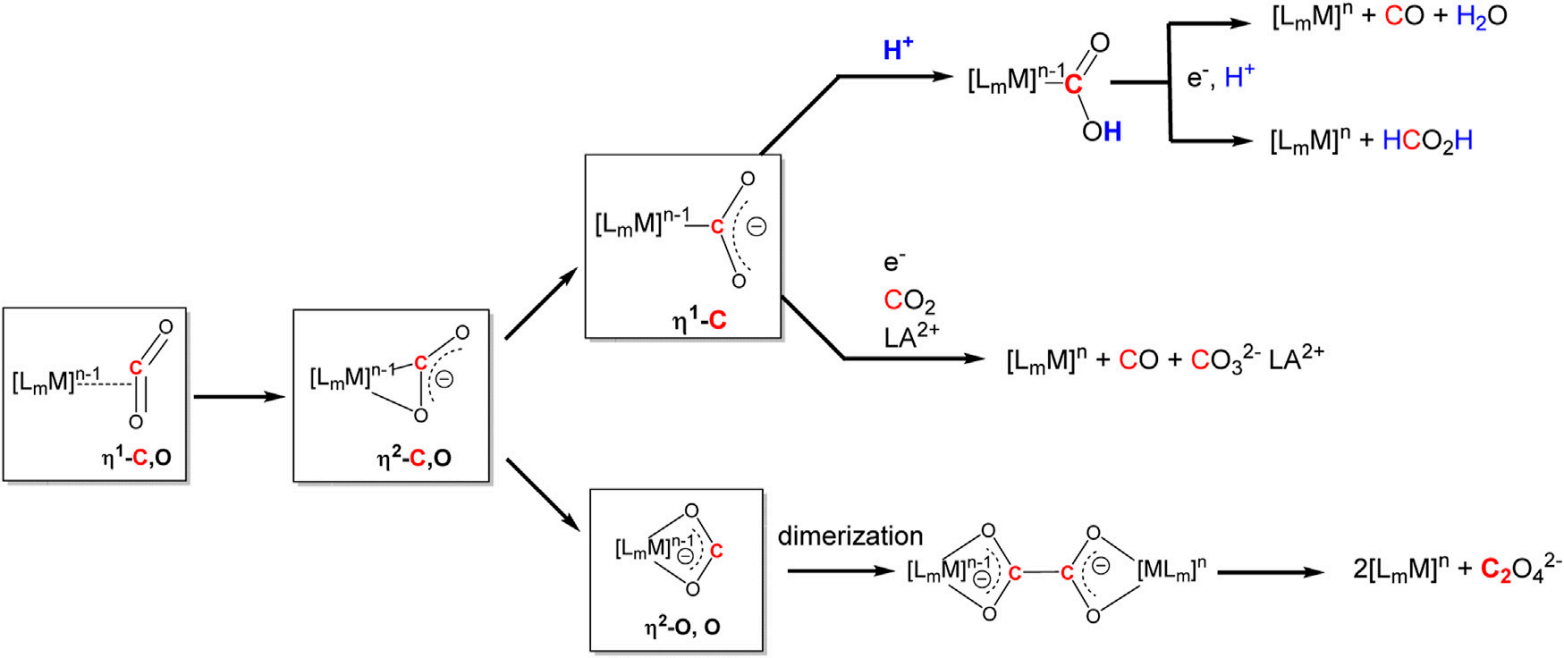
Modification possibilities for CO_2_ activation *via* transition metals. Redrawn from (Grice, 2017; Paparo and Okuda, 2017; Kinzel et al., 2021).

CO_2_ shows a high thermodynamic stability (*ΔG* = −393.5 kJ mol^−1^) (Álvarez et al., 2017). Thus, the compounds must be activated *via* homogenous or heterogeneous catalysis before a transformation can take place at ambient temperature. In the case of electrocatalysis, this activation proceeds *via* surface-catalyzed electro-activation (Álvarez et al., 2017). Transformations of CO_2_ can be initiated *via* electrophilic activation of the oxygen or nucleophilic activation of the carbon atom. Furthermore, direct coordination to the carbon atom to the metal is also possible (Scheme 1). Moreover, CO_2_ can be simultaneously activated by two metal atoms, which allows for the design of highly sophisticated metal complexes as efficient and selective catalysts. (Grice, 2017; Paparo and Okuda, 2017; Kinzel et al., 2021).

The electrodes and more importantly, electrocatalysts, play an important role in the product formation and product distribution. Typically, the electrocatalyst is absorbed at the cathode and interacts with CO_2_ during the electrochemical reduction. Charge transfer proceeds through the interface of the electrode where the catalyst is absorbed, and CO_2_, followed by desorption of the reduced products.

Typically, the reduction commences with the formation of a radical anion (CO_2_^**−**^). This initial step requires a substantial amount of energy (E^0^ = 1.90 V vs NHE, pH 7), as the linear molecule is converted to a bent radical anion. While this initial step is kinetically problematic, subsequent steps are typically energetically favored, driving the reaction to completion. Thermodynamic potentials of common reactions and reaction products are listed in Table 1. Generally, the overall reaction consists of a series of individual steps, including electron and proton transfer reactions (Benson et al., 2009; Zhang et al., 2017a; Khezri et al., 2017; Álvarez et al., 2017).

**TABLE 1:** Selected CO_2_ reduction processes and the corresponding standard redox potentials E^0^ for aqueous solutions (Francke et al., 2018).

**Half-electrochemical thermodynamic reactions**	**E^0^ vs RHE/V**
2H^+^ + 2e^−^ → H_2_ (g)	0.00
CO_2_ (g) + e^−^ → CO_2_^**.**-^	−1,90
CO_2_ (g) + 2H^+^ + 2e^−^→ HCOOH (l)	−0.25
CO_2_ (g) + H_2_O (l) + 2e^−^ → HCOO^−^ (aq) + OH^−^	−1.08
CO_2_ (g) + 2H^+^ + 2e^−^ → CO (g) + H_2_O (l)	−0.11
CO_2_ (g) + H_2_O (l) + 2e^−^ → CO (g) + 2OH^−^	−0.93
CO_2_ (g) + 4H^+^ + 4e^−^ → HCHO (l) + H_2_O (l)	−0.07
CO_2_ (g) + 3H_2_O (l) + 4e^−^ → HCHO (l) + 4OH^−^	−0.90
CO_2_ (g) + 6H^+^ + 6e^−^ → CH_3_OH (l) + H_2_O (l)	+0.02
CO_2_ (g) + 5H_2_O (l) + 6e^−^ → CH_3_OH (l) + 6OH^−^	−0.81
CO_2_ (g) + 8H^+^ + 8e^−^ → CH_4_ (g) + 2H_2_O (l)	+0.17
CO_2_ (g) + 6H_2_O (l) + 8e^−^ → CH_4_ (g) + 8OH^−^	−0.66
2CO_2_ (g) + 2H^+^ + 2e^−^ → H_2_C_2_O_4_ (aq)	−0.50
2CO_2_ (g) + 2e^−^ → C_2_O_4_^2-^ (aq)	−0.59
2CO_2_ (g) + 12H^+^ +12e^−^ → C_2_H_4_ (g) + 4H_2_O (l)	+0.06
2CO_2_ (g) + 12H^+^ +12e^−^ → CH_3_CH_2_OH (l) + 3H_2_O (l)	+0.08

Commonly, electrocatalysts are electron transfer agents, which are operating, in an ideal case, near the thermodynamic potential of the desired reaction. However, in most cases the transformation of CO_2_ proceeds at much higher negative potentials compared to the theoretical ones, and this results in the so-called overpotential. This additionally energy depends on the electrode, the electrolyte, the CO_2_-concentration, the pH value as well as the temperature and the pressure (Benson et al., 2009; Álvarez et al., 2017). This overpotential can be affected by chemical fine-tuning of the ligands of the macrocycle. Moreover, in the presence of an aqueous electrolyte, CO_2_ reduction becomes more challenging due to the competing hydrogen evolution reaction (HER), the low solubility and reactivity of CO_2_ in water, which impedes the transformation. As a consequence, the development of catalysts for electrocatalytic CO_2_ reduction in aqueous environment remains a big challenge. Further research is required to find methods to selectively suppress the formation of hydrogen while simultaneously favoring the reduction of CO_2_ (Khezri et al., 2017; Gonglach et al., 2019).

### Obstacles and Possible Solutions in the Electrochemical CO_2_ Reduction Reaction

The high electrochemical potential required for the reduction of CO_2_ (E^0^_CO2/CO2_ = −1.98 V vs NHE in DMF), triggers a variety of other problems which potentially reduce the overall efficiency of the process. When oxygen is present during the electrochemical reaction, highly reactive intermediates, such as O_2_^−^ or H_2_O_2_ can be formed. These side products are capable of damaging the electrode material or the electrocatalyst by oxidizing or degrading ligands. Therefore, it is important to employ catalysts which are “immune” to oxygen reduction reaction (ORR). Three different approaches have been described in the recent years. One possibility is to introduce a co-catalyst, which quenches any partially reduced oxygen species before damage can occur. Alternatively, a catalyst should be used that reduces oxygen to water. The last method is to use a catalyst which is highly selective towards CO_2_ so that the reduction can take place in presence of O_2_ (Mondal et al., 2019).

## Metal Macrocycles for CO_2_ Electroreduction

The most investigated electrocatalysts suitable to mediate the reductive transformation of CO_2_ are without doubt transition metals and their corresponding metal complexes, respectively. The great potential of these metals can be attributed to their vacant orbitals and active d-electrons, where it is postulated that they favor the formation of an adduct between CO_2_ and the metal and promote afterwards the desorption of reduced products. The metal-type and ligand-structure play an extensive role in their catalytic behavior (Qiao et al., 2014; Kinzel et al., 2021). Generally, molecular catalysts are often less durable compared to solid-materials, since the main reduction product is CO and examples for more than two-electron reduced compounds are rare. Enormous effort was devoted to the preparation of efficient catalysts utilizing earth abundant elements, which address transition metal complexes with the most prominent metals, Fe, Cu, Co, Mn, and Ni (Takeda et al., 2017). Within the past decades, Co or Fe-macrocycles were of great interest for many research groups (Figure 3, compounds **1–11**). Already in the early 1970’s Meshitsuka and co-worker found that Co- and Ni-phthalocyanines electrocatalyze CO_2_ reduction (Meshitsuka et al., 1974; Wang, 2017). Furthermore, in the 1980’s, the research groups of Eisenberg and Sauvage presented the electrochemical reduction of CO_2_ to CO *via* Co− and Ni-tetraazacomplexes with high selectivity (Sauvage: FE 96%, −0.86 V vs SCE) (Fisher and Eisenberg, 1980; Collin et al., 1988; Collin and Sauvage, 1989; Benson et al., 2009; Wang, 2017). In 1998, Saveánt investigated, the Fe(0)porphyrins mediated transformation of CO_2_ to CO in the presence of weak Brønsted acids, such as 1-propanol, 2-pyrrolidine or trifluoromethanol to facilitate the cleavage of one of the C-O bonds in CO_2_. Hydrogen formation could be suppressed, efficiency and lifetime enhanced. Problematic was the use of a mercury working electrode and the quite negative operating potential (−1.5 V vs SCE, DMF) (Bhugun et al., 1996a). Of course, catalytic abilities of diverse other complexes, for instance, with bipyridine or phosphine as ligands were studied which are omitted as this section emphasizes on the application of tetrapyrroles.

The publication of Grodkowski in 2002 has been the only report on CO_2_ reduction with Co− and Fe-corroles for a long time. Stable metal corroles, PPh_3_-Co(III)-TpFPC, Cl-Fe(IV)-TpFPC, Cl-Fe(IV)-TdCC [5,10,15-tris-(2,6-dichlorophenyl)corrole] were studied *via* chemical, electrochemical, and photochemical methods, whereas Co(I) as well as Fe(I) were identified as catalytically active species. As result, the latter Fe-macrocycle showed the highest CO_2_ reduction ability in acetonitrile to form CO. Compared to porphyrins, which can only react with metals with an oxidation state of zero, corroles are able to mediate transformation of CO_2_ with metals with an oxidation state of +I (Grodkowski et al., 2002).

More often, various examples of porphyrins are found as electrocatalysts in the literature. Robert et al. described Fe-porphyrins to be most efficient in aprotic solvents (DMF, ACN), in respect to their catalytic rate, their selectivity and robustness to yield CO. Additionally, Brønsted and Lewis acids improve catalysis as already found by Saveánt. By introduction of positively charged trimethylanilinium moieties into tetraarylporphyrins, CO_2_ to CO conversion is promoted via through-space substituent effects, resulted in a turnover frequency (TOF) of 10^6^ s^−1^ with a low overpotential of 0.220 V and a selectivity of 100% (CO) and stability over 84 h long-term electrolysis (Azcarate et al., 2016a; Takeda et al., 2017).

### CO_2_ Reduction With Metal Porphyrin Complexes

**FIGURE 4:**
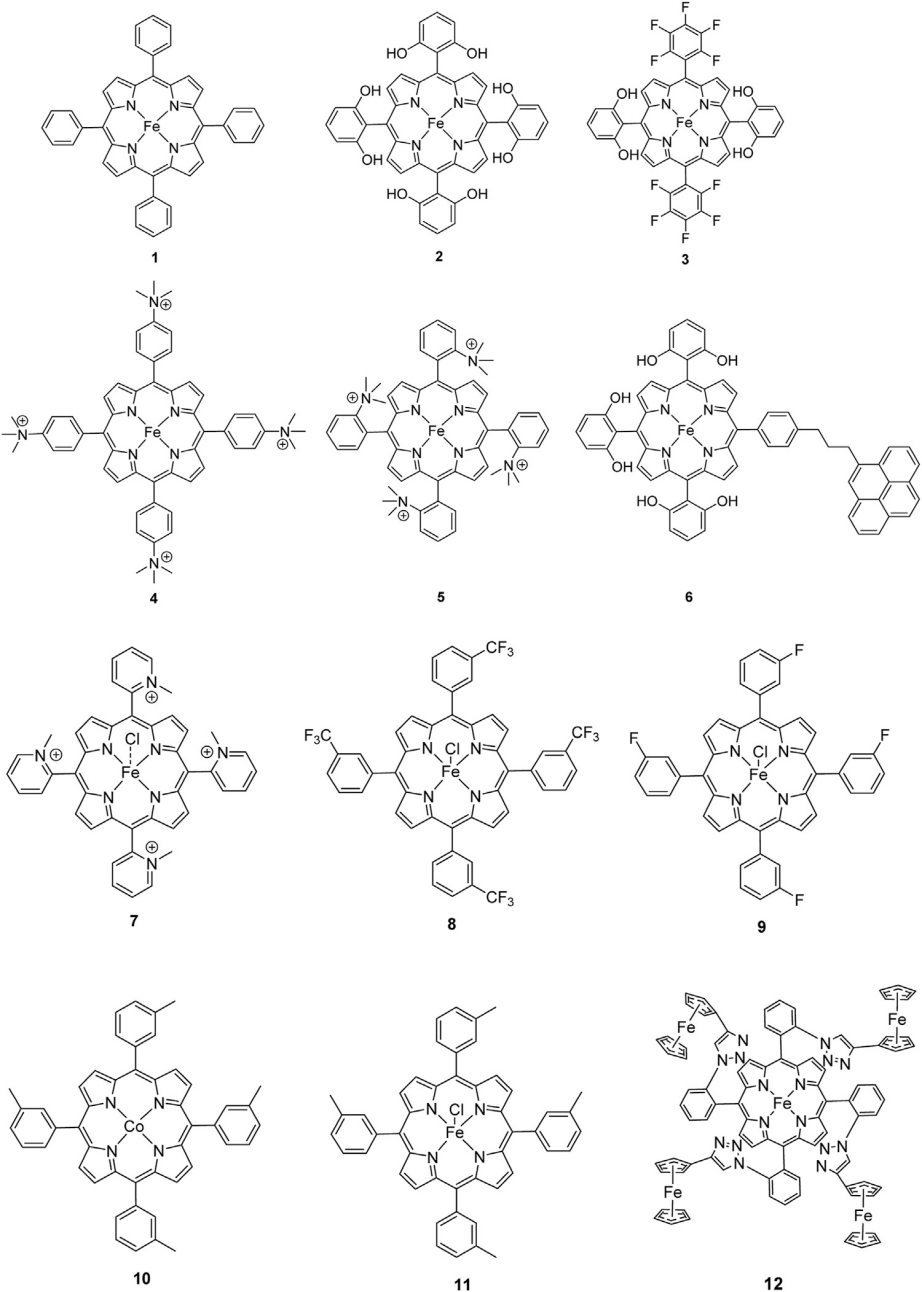
Examples of Fe− and Co-Porphyrin complexes for CO_2_ reduction to CO (Mondal et al., 2019).

Iron porphyrins, illustrated in Figure 4, were first described in photocatalytic systems for CO_2_ reduction by Neta et al. in 1997 (Grodkowski et al., 1997). Photoexcitation of the ligand-to-metal charge transfer (LMCT) absorption band at 360 nm of a DMF/TEA (5%) solution containing compound **1** with an axial chloride ligand caused a one-electron reduction of the central metal from Fe^III^ to Fe^II^, simultaneously releasing the chloride ligand. The Fe^II^ species could be further reduced to Fe^I^ by triethylamine (TEA). Disproportionation of two Fe^I^ molecules produces the catalytically active Fe^0^ species, which coordinates to CO_2_. The product of the CO_2_ reduction was CO with a turn-over-number of the reaction of TON_CO_∼70, and H_2_ was formed as a minor product. During the catalytic process, photo-Birch reduction of the porphyrin ring occured, converting it to the corresponding chlorin structure, followed by further photochemical decomposition.

The cationic Fe porphyrin **7** has also been reported to function as a photocatalyst, generating CO from CO_2_ by photoexcitation in an aqueous solution (pH 8.8) containing TEA as sacrificical electron-donor (SD) and NaHCO_3_. Because the efficiencies of the photochemical reduction of the Fe porphyrins were very low, the CO_2_ photoreduction proceeds with extremely low efficiencies. Addition of *p*-terphenyl as a photosensitizer (PS) to photocatalytic systems using Fe porphyrins (FeP), was investigated in order to overcome the above mentioned problems (Dhanasekaran et al., 1999). The standard reduction potential of PS-OERS of the PS was negative enough (−2.45 V vs SCE in dimethylamine) to reduce the Fe^II^P species −1.05 V vs SCE for **1**, −1.02 V vs SCE for **8**, and −1.00 V vs SCE for **9** and Fe^I^P (−1.66 V vs SCE for **1**,−1.61 V vs SCE for **8**, and −1.55 V vs SCE for **9**) so as to form the corresponding Fe^0^P species. This system exhibited 10 times higher photocatalytic efficiencies than those measured in the absence of the PS. The Co porphyrin **10** can also be used to produce CO, and its photocatalytic efficiency was about 1.5 times higher compared to that of the corresponding Fe porphyrin (**11**, Table 2; Dhanasekaran et al., 1999).

**TABLE 2:** Comparison of catalyst systems, major products, maximum FEs, and mechanisms of macrocyclic complexes in electrochemical CO_2_ reduction (n.a., not available; prop., proposal; comp., computational investigation; exp., experimental evidence).

Entry	Catalyst systems	Substitution(s)	Major product	Max.FE (%)	Mechanism	Basis	Method	References
**Porphyrins**								
1	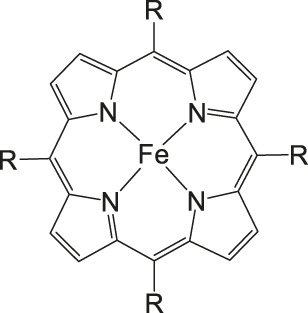	*R* = Ph, C_6_F_5_, pyren-1-yl,	CO/H_2_O	100	ET_M_	Comp.	DFT	Hammouche et al. (1988), Hammouche et al. (1991), Bhugun et al. (1994), Bhugun et al., (1996a), Bhugun et al. (1996b), Costentin et al. (2013), Costentin et al. (2015), Azcarate et al. (2016a), Ambre et al. (2016), Azcarate et al. (2016b), Choi et al. (2016), Okabe et al. (2017), and Margarit et al. (2020a)
		meso-thien-2-yl,	HCO_2_^−^	72				
		meso-5-methylthien-2-yl						
2	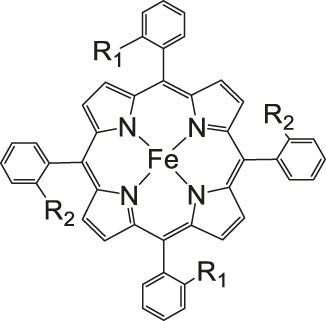	R_1_ = R_2_ = CO_2_Me,	CO/H_2_O	100	ET_M_	Comp. Ambre et al. (2016), and Sen et al. (2019)	DFT	Hammouche et al. (1988); Hammouche et al. (1991), Azcarate et al. (2016a), Ambre et al. (2016), Khadhraoui et al. (2018), Mondal et al. (2019), and Sen et al. (2019)
		NHCOtBu,						
		NHCOC_6_H_4_CH_2_MeIm^+^,						
		NMe_3_						
		+, trFc_2_, trCO_2_Me,						
		tr-4-*t*Bu						
		R_1_\R_1_/R_2_\R_2_ = NHCO-						
		(CH_2_)_10_CONH, NHCO-						
		(CH_2_)_10_ImCONH						
3	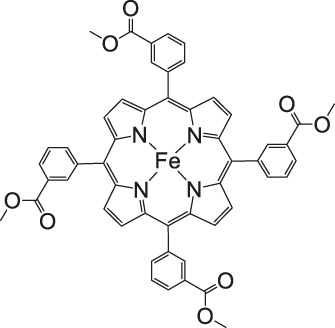	-	CO/H_2_O	65	ET_M_	Comp.	DFT	Ambre et al. (2016)
4	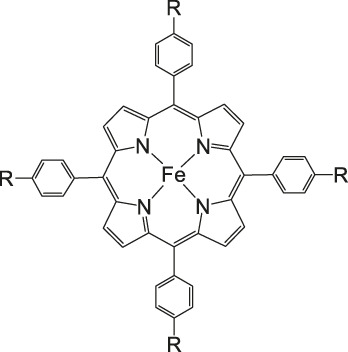	*R* = Ph, pyren-1-yl,	CO/H_2_O	100 (66)	ET_M_	Comp.	DFT	Costentin et al. (2015), Azcarate et al. (2016a), Ambre et al. (2016), Tatin et al. (2016), Okabe et al. (2017), and Torbensen et al. (2020)
		CO_2_Me, NMe_3_^+^, SO_3_^−^	H_2_	84 (67)				
5	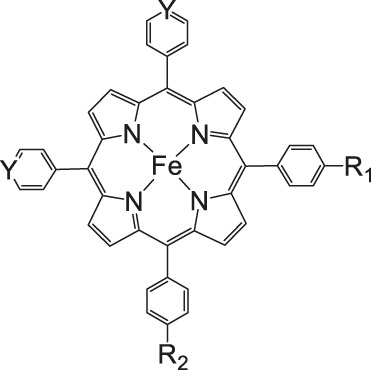	*R*_*1*_ = CH_2_CONHC_6_H_3_-	CO/H_2_O	90 (79)	ET_M_	Comp.	DFT	Nichols et al., (2018), Abdinejad et al. (2019), Abdinejad et al., (2020a), and Abdinejad et al. (2020b)
		(CF_3_)_2_, NHCOCH_2_C_6_H_3_-	CH_4_	41 (80)				
		(CF_3_)_2_,						
		NHCONH-Fe-TPP,						
		OMe						
		*R*_*2*_ = -H, -NH_2_, -OMe						
		*Y*= CH, N						
6	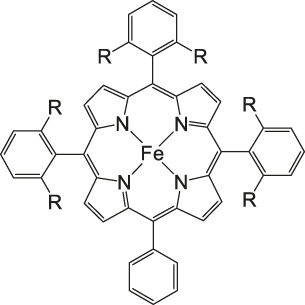	*R* = -OH, -OMe	CO/H_2_O	94 (83)	ET_M_	Prop.	n.a.	Costentin et al. (2012), Costentin et al. (2015), and Azcarate et al. (2016b)
7	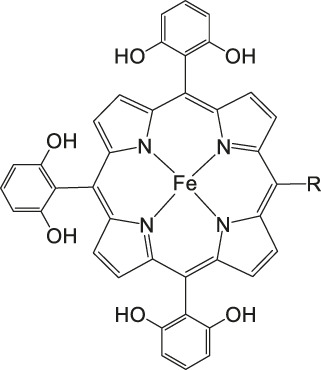	*R* = -propylpyrene	CO/H_2_O	97	n.a.	n.a.	n.a.	Maurin and Robert (2016)
8	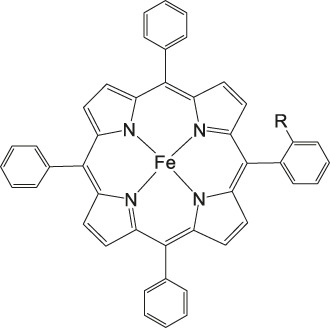	*R* = -CH_2_CONHC_6_H_3_-	CO/H_2_O	96 (85)	ET_M_	Comp.	DFT	Nichols et al. (2018) and Sinha and Warren (2018)
		(CF_3_)_2_, NHCOCH_2_C_6_H_3_(CF_3_)_2_,						
		-OH						
9	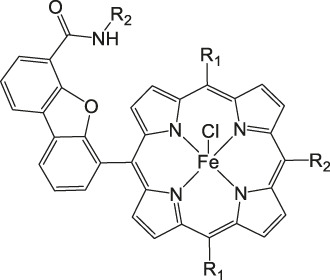	*R*_*1*_ = 3,4,5-trimethoxyphenyl	CO/H_2_O	96	ET_M_	Comp.	DFT	Margarit et al. (2019)
		*R*_*2*_ = CNHNH_2_,						
		-C_6_H_4_OH, -C_6_H_4_SO_3_H						
10	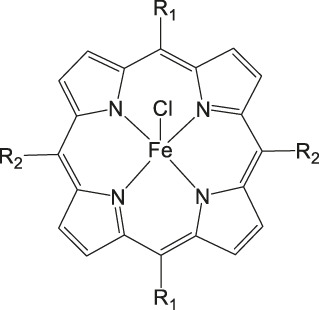	*R*_*1*_ = 3,4,5-trimethoxyphenyl	CO/H_2_O	100	ET_M_	Prop.	n.a.	Margarit et al. (2020b)
		*R*_*2*_ = 3,4,5-trimethoxyphenyl						
		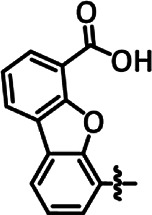						
11	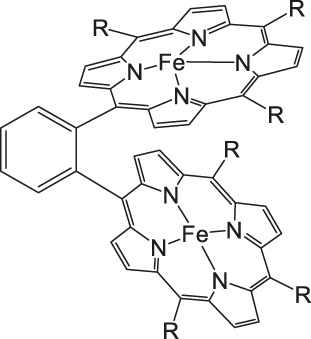	*R* = Ph, Me_3_C_6_H_2_, C_6_F_5_,	CO/H_2_O	92 (88)	ET_M_	Prop. Zahran et al., (2016)	n.a.	Mohamed et al. (2015) and Zahran et al. (2016)
		2,6-Cl_2_C_6_H_3_, 2,6-F_2_C_6_H_3_						
12	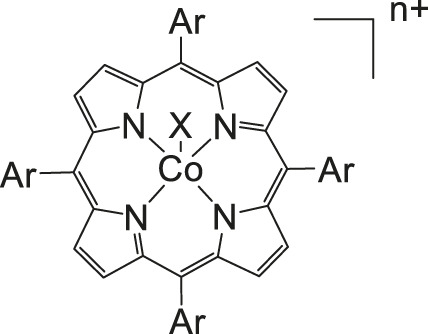	Ar=Ph, C_6_H_4_CF_3_, C_6_H_4_NH_2_,	CO/H_2_O	97 (90)	ET_M_	Prop. Hu et al., (2017)	n.a.	Behar et al. (1998), Lin et al. (2015), Alenezi (2016), Hu et al. (2017), Zhu et al., (2019), Hu et al. (2020), and Jack et al. (2020)
		C_6_H_4_NMe_3_^+^, C_6_H_3_(OH)_2_,						
		C_6_H_4_OMe, C_6_H_3_(OMe)_2_,						
		C_6_H_4_Cl, C_6_H_4_Br, C_6_H_4_F, C_6_F_5_						
		*n* = 0, 1						
		X = -Cl						
13	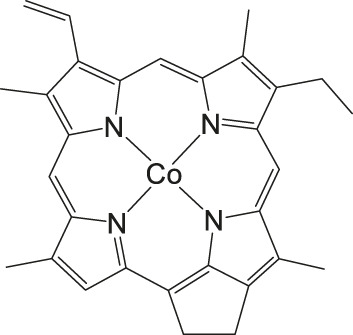	-	CO/H_2_O	89	ET_M_	Prop.	n.a.	Aoi et al. (2015)
**Phthalocyanines**								
14	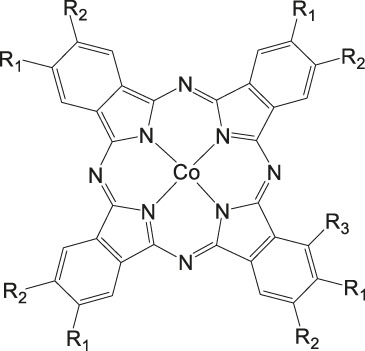	*R*_*1*_ = H, *t*Bu, OC_8_H_17_, O-py	CO/H_2_O	100 (60)	ET_M_	Exp.	PSCAS	Mahmood et al. (1987), Abe et al. (1996), Han et al. (2017), Zhang et al. (2018), Wang (2019b), Choi et al. (2019), Liu and McCrory (2019); Ren et al. (2019), Boutin et al. (2020), Hu et al. (2020), and Riccardis et al. (2020)
		*R*_*2*_ = H, *t*Bu, OC_8_H_17_, O-py	H_2_	72 (60)		Comp.	DFT	
		*R*_*3*_ = H, NMe_3_	MeOH	MeOH(20)				
		^+^						
15	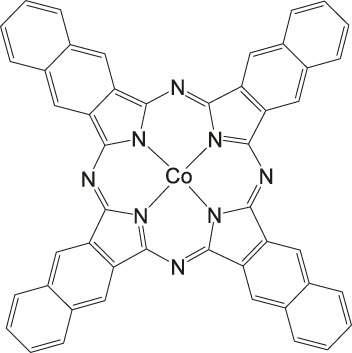	-	CO	97	n.a.	n.a.	n.a.	Wang et al. (2019a)
16	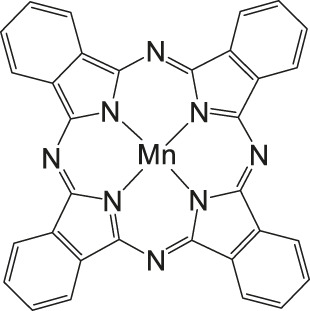	-	HCOO^−^	26	n.a.	na.	na.	Mahmood et al. (1987)
			H_2_	77				
17	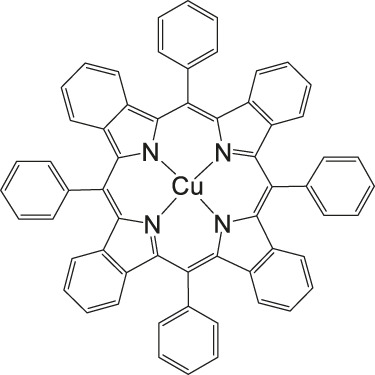	-	CO	48	n.a.	n.a.	n.a.	Apaydin et al. (2018)
18	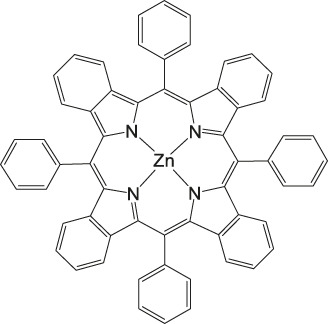	-	CO	33	n.a.	n.a.	n.a.	Apaydin et al. (2018)
**Corroles**								
19	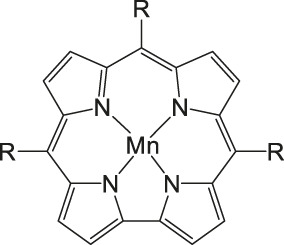	*R* = -C_6_F_4_-S-(PEG7)-OCH_3_	CH_3_OH	23	ETM	Exp.	EAS, [b] GCMS,	De et al. (2020)
			CH_3_CO_2_^−^	63		comp.	IL, IR-,	
			_				UV/Vis-	
							SEC, NMR	
							DFT	
20	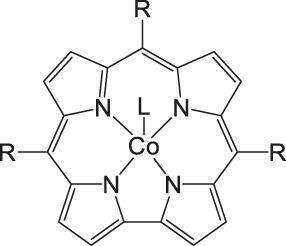	*L* = -PPh_3_	HCO_2_^−^	12				Gonglach et al. (2019)
		*R* = -C_6_F_5_-S-(PEG7)-OCH_3_	CH_3_OH	59				
			HCOH	10				
			CH_3_CH_2_OH	48				
			CH_3_CO_2_^−^	13				
			H_2_	36				
21	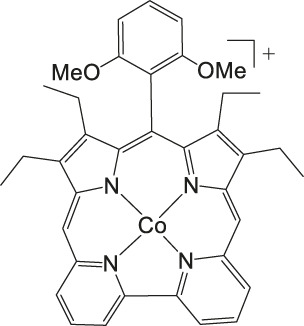	-	CO/H_2_O	77	n.a.	n.a.	n.a.	Ogawa et al. (2019)

A promising candidate for this particular reduction is the FeFc_4_-porphyrin complex **12**, which has four ferrocene moieties attached and a bromide as an axial ligand (Figure 4). This particular catalyst has the ability to catalyze the reduction of O_2_ to H_2_O when the oxidation state is Fe(II) (4H^+^/4e^−^) and reduce CO_2_ to CO (2H^+^/2e^−^) in the presence of Fe (0). During ORR three of four ferrocenes are oxidized together with the Fe(II) center, therefore providing the four electrons to conduct the ORR (Mondal et al., 2019). In this experiment, phenol served as proton source. The two forms, Fe(II) in presence of Fe(0), are obtained by choosing two different onset-potentials to achieve the desired catalyzed reaction. Those onset-points are determined through cyclic voltammetry and the cyclic voltametric responses. In case of the FeFc_4_-porphyrin complex the needed potentials are 0.0 V to −0.67 V for the ORR and −2.50 V for CO_2_RR. Overall, the CO_2_ reduction to CO happens with a Faradaic efficiency of >92%.

The selectivity of this porphyrin complex is confirmed by inducing the CO_2_ reduction in the presence of O_2_ and maintaining the catalytic current for the CO_2_RR at −2.50 V even when the pressure of O_2_ is increased. Bulk electrolysis with 20% O_2_ shows that only 5–6% partially reduced oxygen species are found, produced by this porphyrin-complex when the oxidation state is Fe(II). However, the oxidation of the Fe(0) porphyrin-complex by O_2_ happens rather unlikely, given that the Faradaic yield for the CO_2_RR is 43% when a 1:3 mixture of CO_2_:O_2_ is induced. The selectivity of the Fe (0) porphyrin-complex for CO_2_RR over ORR can be argued in different ways. Thermodynamically speaking, the formation of Fe(II)-O_2_^2−^ is favored over Fe(II)-CO_2_^2−^ with a difference in absolute free energy of around 22.68 kcal/mol, showing that thermodynamics are not the reason for the preference. On the other hand, reaction of the complex with both gases separately shows in absorption spectroscopy that the specific Soret-band for porphyrins shifts immediately after inducing CO_2_ while the reaction with O_2_ shows little to no change. Hence, the kinetic barrier for the reaction with CO_2_ is lower, making the CO_2_RR 500 times faster when pseudo first-order kinetics is assumed. Involving the different solubilities of the gases in acetonitrile (CO_2_ = 0.28 M and O_2_ = 0.01 M), one can determine that the rate constants for the first- and second-order are one magnitude higher for CO_2_ reduction (Mondal et al., 2019).

### CO_2_ Reduction Reaction With Metal Phthalocyanine Complexes

**FIGURE 5:**
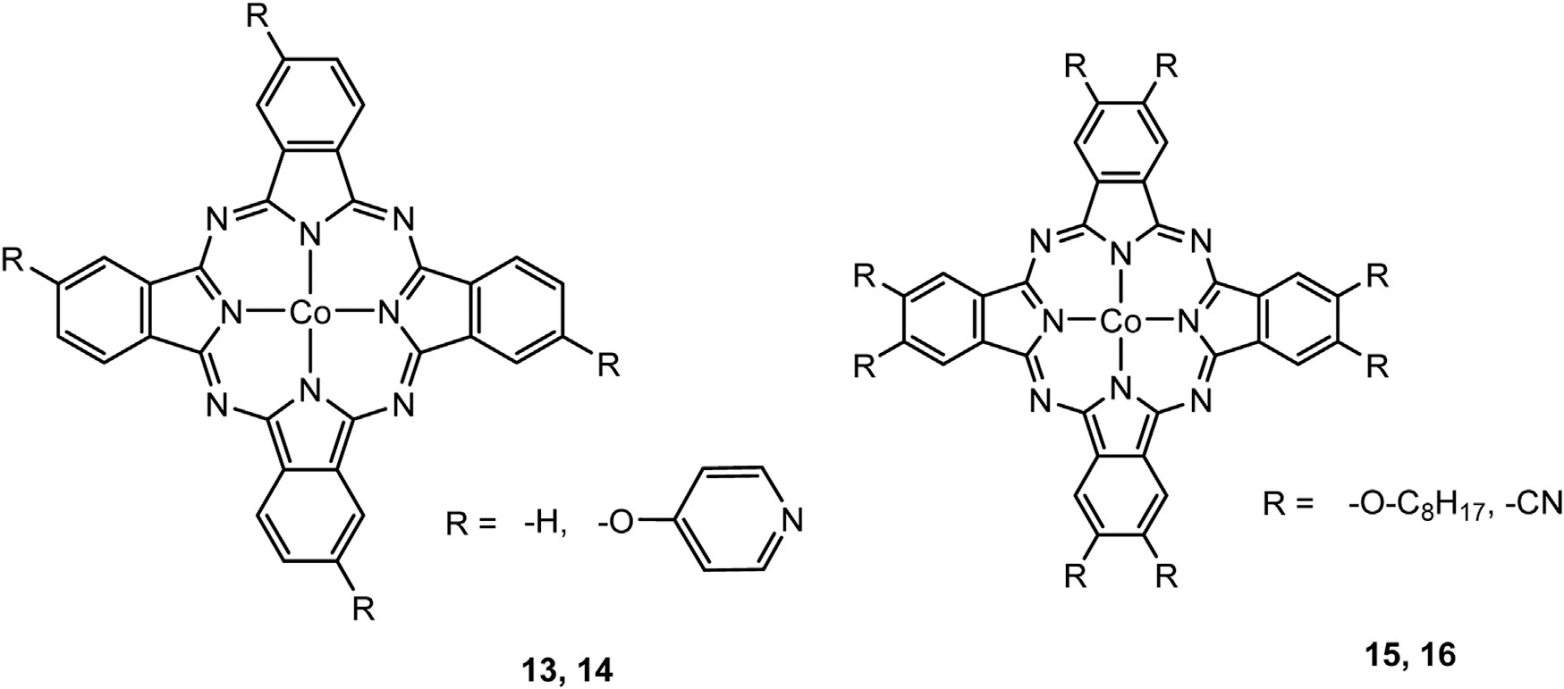
Co− and Fe phthalocyanine complexes for CO_2_RR (Zhang et al., 2017b).

As mentioned in previous sections, a suitable catalyst for the CO_2_RR should be selective to CO_2_ or in other words be “immune” against the hydrogen evolution reaction HER, should have a high chemical and thermal stability and be inexpensive in production. Therefore, catalysts such as porphyrins or phthalocyanines using non-noble metals and having all the above-mentioned properties are the center of investigations. (Zhang et al., 2017a) In one described case, De Riccardis et al. investigated a phthalocyanine complex metalated with Co and with pyridine as a peripheral substituent (Figure 5). The second electrochemical reduction of this complex Co(II)Pc-Pyr happens at −0.4 V vs. Ag/AgCl, resulting in a catalytically active [Co(I)Pc-Pyr^−^]^−2^ species. The Faradaic efficiency for CO reaches a total of 95% at this specific onset-potential. Such a high FE is achieved through the inductive effect of the pyridine moieties, which supports the CO_2_ adsorption and increases the electron affinities of the metal center. In comparison to the non-substituted CoPc complex that only has 80% FE at −0.6 V vs RHE for the conversion of CO_2_ to CO and polycrystalline Ag requiring at least −0.7 V to conduct the same reduction, the CoPc-Pyr shows to be a promising candidate for CO_2_RR. Additionally, at an onset-potential of—0.7 V vs RHE, the turnover frequency of this phthalocyanine complex is around 6.9 s^−1^ when 10^−8^ mol are present (Riccardis et al., 2020).

Other examples of Mn-based molecular catalysts capable of formate formation during electrochemical CO_2_ reduction are the systems reported by Mahmood et al. The Mn–phthalocyanines synthesized by this research group exhibited an FE of 26% at −2.00 V vs. saturated calomel electrode (SCE) after attachment to a PTFE-bonded carbon gas diffusion electrode. Hydrogen generation (FE = 77%) exceeded the formation of formic acid, but no further mechanistic investigations were pursued (Mahmood et al., 1987).

### CO_2_ Reduction Reaction With Metal Corrole Complexes

**FIGURE 6:**
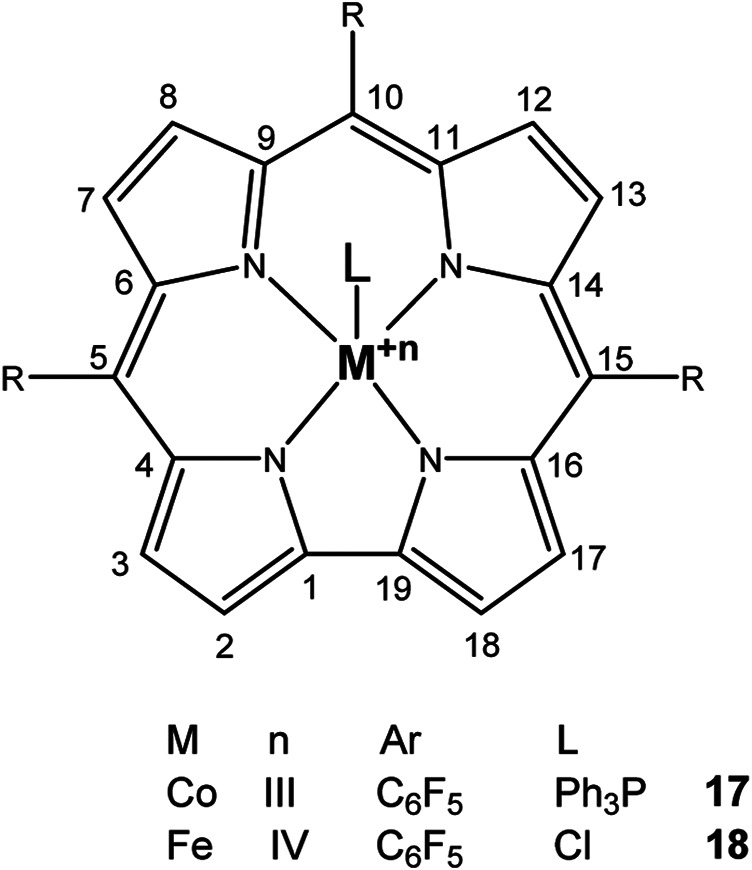
Co and Fe corroles for the homogeneous CO_2_ reduction to CO and H_2_ in MeCN solutions.

Metal corroles are structural similar to metal porphyrins with both the metal centers and ligands participating in multielectron redox processes and are promising candidates for efficient proton-coupled electron transfer (Zhang et al., 2017b; Hu et al., 2017; Shen et al., 2015). These metal complexes stabilize radical intermediates thus providing an effective pathway to facilitate C–C step-up. (Behar et al., 1998; Kortlever et al., 2015) Cobalt and iron corroles have been previously found to be catalytically active for CO_2_ reduction to CO (Figure 6; Grodkowski et al., 2002).

**FIGURE 7:**
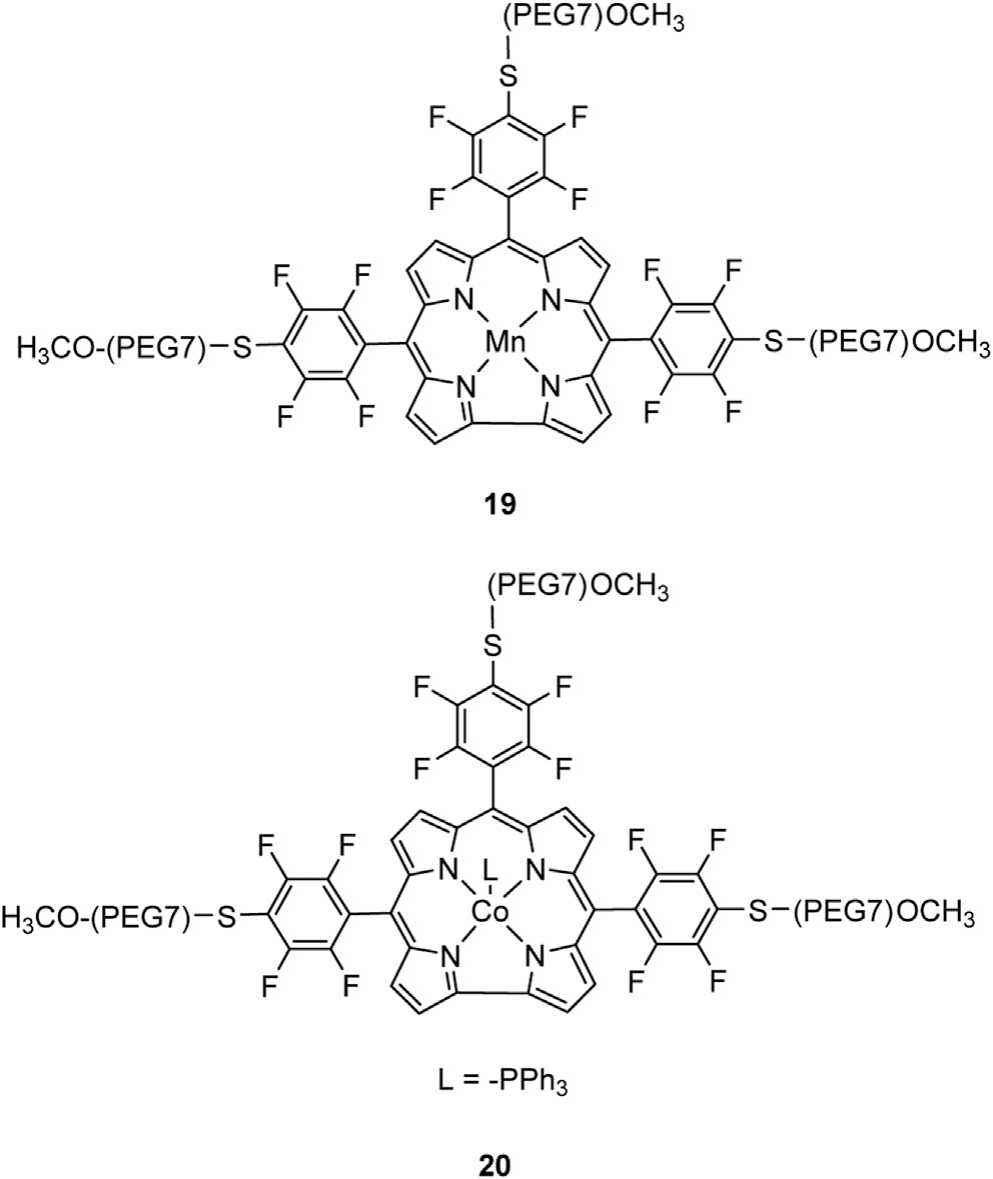
Co− and Mn-corrole complexes reported by Roy and Schöfberger et al. (Gonglach et al., 2019).

Our group recently reported a polyethylene glycol (PEG)-modified Mn–corrole complex immobilized on a carbon paper electrode, as an example of a manganese catalyst capable of producing MeOH (FE = 23%) and acetate (FE = 63%). (De et al., 2020) Although a detailed mechanistic investigation has not yet been performed, the authors propose an ETM pathway with a possible Mn(III) carboxyhydroxyl intermediate towards methanol formation and an oxalate type key species for acetate production. As another example for immobilized Co macrocycles in electrochemical CO_2_ reduction, the polyethylene glycol derivatized Co–corrole (Figure 7) reported by Gonglach et al. showed remarkable catalytic activity when used on carbon paper electrodes (Gonglach et al., 2019). More specifically, it produces ethanol and methanol in a Faradaic efficiency of 47 and 59%, respectively, at −0.73 V vs. reverse hydrogen electrode. Employment of GC‐MS (in combination with ^2^D‐ and ^13^C‐labeling), NMR, EPR, IR‐SEC, and complementary control experiments resulted in the proposed mechanism(s).

## Organic Inorganic/Polymer Hybrid Materials for CO_2_ Reduction

**FIGURE 8:**
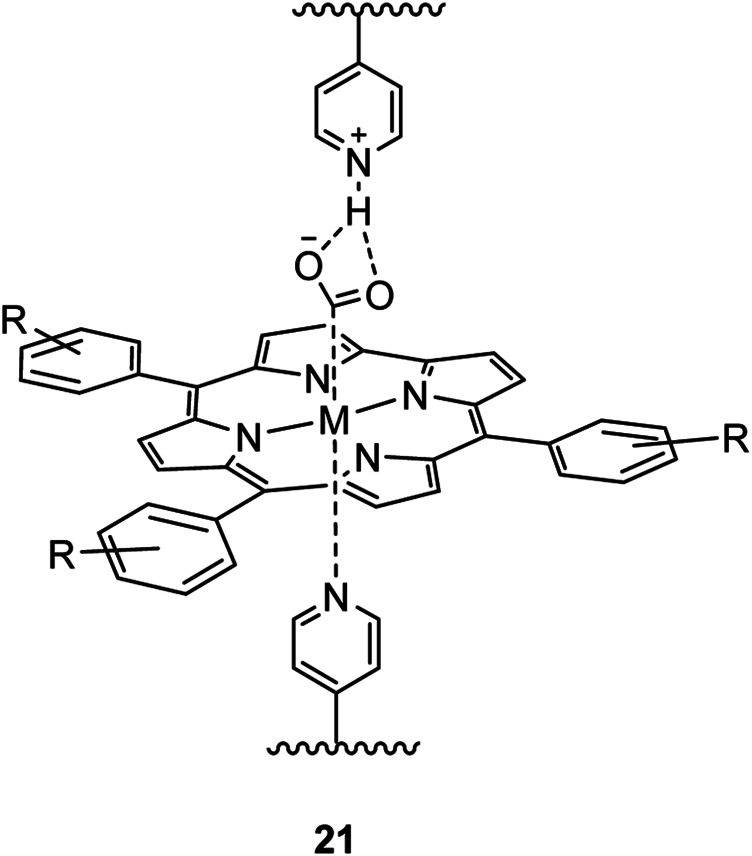
Immobilization of CoPc catalysts on P4VP (Kramer and McCrory, 2016).

Organic–inorganic hybrid materials, which are usually defined as multicomponent compounds containing organic (biological) and inorganic components in the sub-micrometric and nanometric scale can be used to integrate excellent properties, such as selectivity (Sanchez et al., 2005). In general, the organic and inorganic components can be linked by noncovalent bonds (such as van der Waals, hydrogen bonds, or electrostatic bonds) and/or covalent bonds in hybrid systems. This kind of materials not only effectively combine the advantages of organic materials (variety, flexibility, etc.) and inorganic materials (large surface area, conductivity, etc.) (Judeinstein and Sanchez, 1996; Faustini et al., 2018; Zhang et al., 2019; Rebber et al., 2020; Yang et al., 2020), but also improve the physicochemical properties, such as the increased CO_2_ adsorption and conductivity, enriched types of active sites, maximized exposure of active sites, and manipulated reaction pathways by tuning the stability of intermediates, thereby synergistically reducing the overpotential and promoting product selectivity (Nam et al., 2020; Sun et al., 2020). As an example, Kramer et al. grafted a cobalt metalated phthalocyanine complex onto a pyridine-substituted polymer that resulted in an increase of selectivity towards CO due to an axial coordination between carbon monoxide and the metal center (Kramer and McCrory, 2016). Immobilization of cobalt phthalocyanine in poly-4-vinylpyridine dramatically improves its activity as a catalyst for the reduction of CO_2_ to CO (Figure 8). The polymer membrane slows the competing HER catalytic pathway while also increasing rate of CO_2_RR compared to the polymer free catalyst.

Liang, Wang and co-workers showed that immobilizing the catalyst onto carbon nanotubes decreases the measured overpotential (Jiang et al., 2019). The group prepared a series of metal (Co, Fe, Mn) phthalocyanine/carbon nanotube hybrids and study their catalytic performance for CO_2_ electroreduction. Both CoPc/CNT and FePc/CNT are active catalysts to reduce CO_2_ to CO and are able to deliver a reduction current density of −1.0 mA/cm^2^ above −0.47 V. FePc/CNT is just slightly less active than CoPc/CNT, but superior to CoPc/CNT with higher FEs for CO at low overpotentials. Manthiram and coworkers suggest that loading catalysts onto electrodes could have a positive impact on the TOF.

**FIGURE 9:**
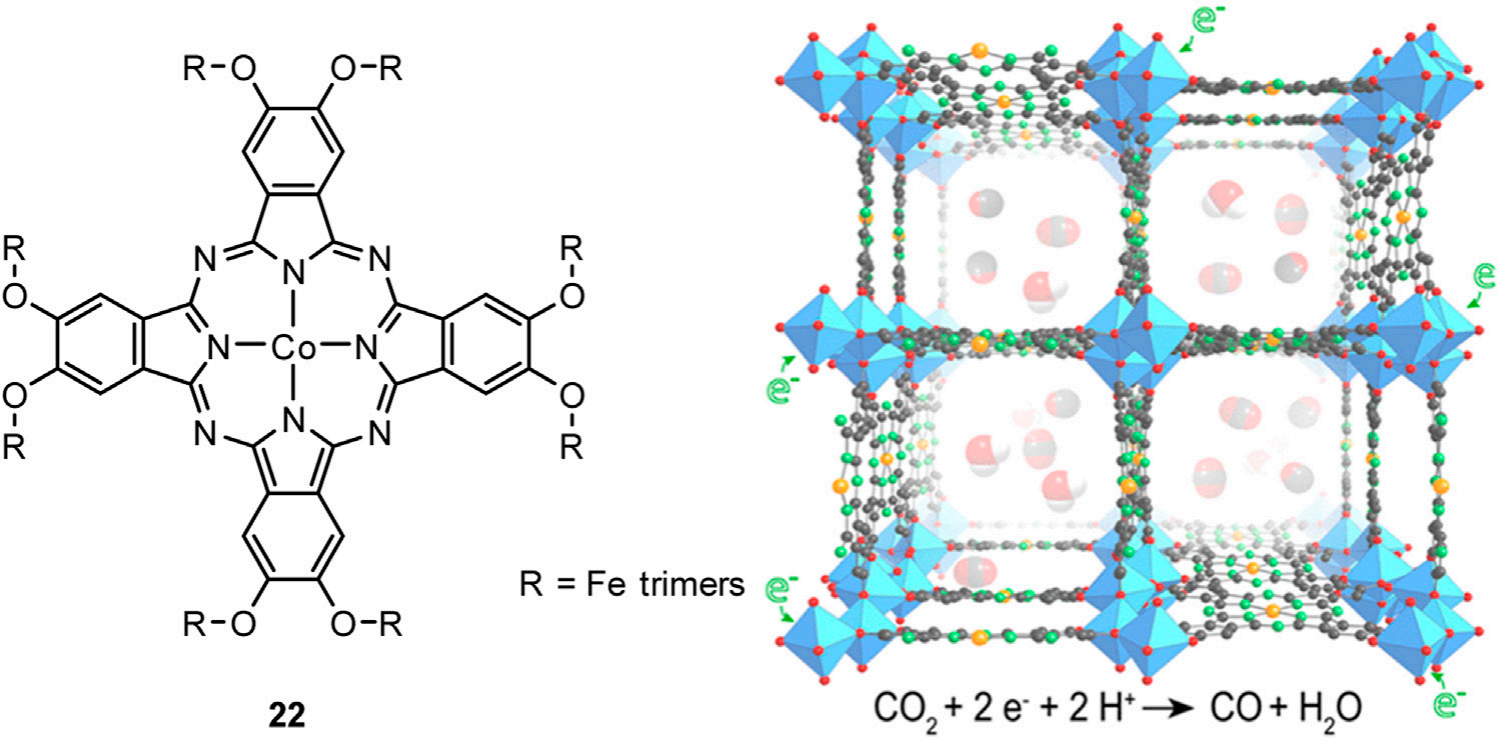
CoPc with Fe_3_ (-C_2_O_2_-)_6_(OH_2_)_2_ trimers **22** forming a 3D metal-catecholate framework. [Reprinted with permission from Matheu et al. (2019)].

Another observation of Chio et al. showed that alkoxy substituents on a cobalt phthalocyanine suppress the aggregation of the complex on graphene sheets *via* π-π stacking and enhance the catalytic activity per single CoPc-A molecule, resulting in FE of 75% at an overpotential of 480 mV. (Choi et al., 2019) An attractive class of solids for the CO_2_RR are metal-organic frameworks (MOFs), which can be used to build porous extended structures. Matheu et al. have shown that the active sites on Co are sterically accessible when applied on a 3D metal-catecholate framework/carbon black cathode materials. The 3D metal-catecholate framework was synthesized by linking tetratopic cobalt phthaloocyanin-2, 3, 9, 10, 16, 17, 23, 24-octanol linkers with Fe_3_ (-C_2_O_2_-)_6_(OH_2_)_2_ trimers. (Matheu et al., 2019) These cathodes based on MOF-1992 and carbon black (CB) display a high coverage of electroactive sites (270 nmol cm^−2^) and a high current density (−16.5 mA cm^−2^; overpotential, −0.52 V) for the CO_2_ to CO reduction reaction in water (faradaic efficiency, 80%). Over the 6 h experiment, MOF-1992/CB cathodes reach turnover numbers of 5,800 with turnover frequencies of 0.20 s^−1^ per active site (Figure 9).

## Conclusion

In this mini-review, we have discussed mechanistic details of various porphyrinoid based catalyst systems during the (photo-)-electroelectrochemical CO_2_ reduction reaction. We have compared the electrocatalytic performance and product distribution of a variety of iron, manganese and cobalt porphyrin, corrole, and phthalocyanine complexes. The reduction efficiency of carbon dioxide by different metal porphyrinoid systems based materials can be extracted from Table 1. One of the future research directions will focus on how to regulate and modify the structure of porphyrinoids. In addition to the efficient reduction of carbon dioxide, it is necessary to consider whether the reduction products can be efficiently further employed. Next to this, one has to consider if the catalyst materials can be synthesized environmentaly friendly.

Advances in experimental techniques have revealed a wealth of mechanistic information in recent years, yet detailed operando-spectroscopic, thermodynamic and kinetic studies of proton and CO_2_ binding to the reduced metal catalysts would help to understand the mechanisms for CO_2_ reduction. Groundbreaking research has to be continued to produce renewable fuels (CO, and ultimately further reduced species such as methanol, methane, ethanol acetic acid etc.) *via* low-energy pathways using durable and selective earth-abundant catalysts for creating carbon-neutral energy sources.
